# A Robust Blood Vessel Segmentation Technique for Angiographic Images Employing Multi-Scale Filtering Approach

**DOI:** 10.3390/jcm14020354

**Published:** 2025-01-08

**Authors:** Agne Paulauskaite-Taraseviciene, Julius Siaulys, Antanas Jankauskas, Gabriele Jakuskaite

**Affiliations:** 1 Artificial Intelligence Centre, Faculty of Informatics, Kaunas University of Technology, 51423 Kaunas, Lithuania; julius.siaulys@ktu.edu; 2 Centre of Excellence for Sustainable Living and Working (SustAInLivWork), 51423 Kaunas, Lithuania; antanas.jankauskas@lsmu.lt (A.J.); gabriele.jakuskaite@lsmu.lt (G.J.); 3 Faculty of Medicine, Lithuanian University of Health Sciences, 44307 Kaunas, Lithuania

**Keywords:** vessel segmentation, computer vision, deep learning, annotation, predictions, Duck-Net

## Abstract

**Background**: This study focuses on the critical task of blood vessel segmentation in medical image analysis, essential for diagnosing cardiovascular diseases and enabling effective treatment planning. Although deep learning architectures often produce very high segmentation results in medical images, coronary computed tomography angiography (CTA) images are more challenging than invasive coronary angiography (ICA) images due to noise and the complexity of vessel structures. **Methods**: Classical architectures for medical images, such as U-Net, achieve only moderate accuracy, with an average Dice score of 0.722. **Results**: This study introduces Morpho-U-Net, an enhanced U-Net architecture that integrates advanced morphological operations, including Gaussian blurring, thresholding, and morphological opening/closing, to improve vascular integrity, reduce noise, and achieve a higher Dice score of 0.9108, a precision of 0.9341, and a recall of 0.8872. These enhancements demonstrate superior robustness to noise and intricate vessel geometries. **Conclusions**: This pre-processing filter effectively reduces noise by grouping neighboring pixels with similar intensity values, allowing the model to focus on relevant anatomical structures, thus outperforming traditional methods in handling the challenges posed by CTA images.

## 1. Introduction

### 1.1. Gaps and Objectives

Vascular segmentation is a very important topic in medical image analysis [1,2]. Accurate vascular imaging is essential for diagnosing disease, planning and applying treatment, and assessing clinical outcomes in a wide range of medical fields, including cardiology, laryngology, ophthalmology, oncology, radiology, and more [3,4,5,6,7]. In addition, it plays an important role in areas of medicine where vascular status has a direct impact on disease progression and treatment. The analysis of vascular imaging is particularly crucial for the diagnosis, treatment, and management of cardiovascular diseases, including stenoses and other vascular abnormalities. Segmentation results can provide detailed information on the structure, shape, and flow dynamics of the vascular system, which is essential for the detection and evaluation of conditions such as arterial blockages, aneurysms, and other pathologies [8,9,10]. Particularly in the case of coronary stenosis, accurate segmentation of the blood vessels allows physicians to determine the degree of narrowing, assess the circulatory disturbances, and plan interventions such as stent placement or bypass surgery. In addition, segmentation allows for a more accurate determination of whether additional diagnostic tests, such as contrast imaging or other advanced techniques, are necessary, ensuring that invasive procedures are performed only when required.

**Figure 1:**
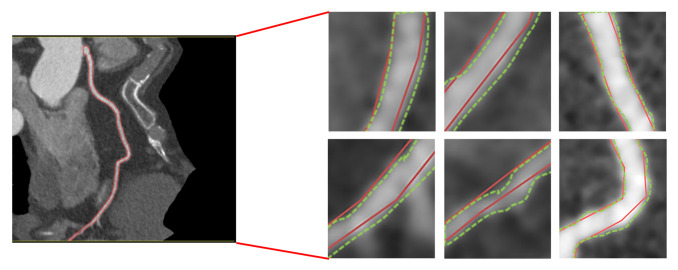
Examples of precise contouring challenges in medical imaging of blood vessels.

Accurate segmentation is essential for reliable diagnosis. However, this task has many challenges, including the complex and intricate structure of blood vessels, the presence of noise in medical images, and the large variation in blood vessel size, shape, and contrast [11,12,13]. This noise can come from a variety of sources, including the imaging process itself, making the task even more complex [14]. It is also important to note that the geometry of coronary vessels can be complex, with curves and bends, requiring the use of algorithms that can precisely trace their path [15]. Inaccuracies can also be due to human error, which can be caused by fatigue, tool sensitivity, and other factors, as observed in Figure 1. These errors directly affect the accuracy of the segmentation models and even raise questions about what the ground truth is—the annotation of the physician or the tool.

### 1.2. Research Contributions and Novelty

Currently, existing methods for vessel segmentation are generally divided into traditional image processing techniques and modern machine learning-based approaches. Traditional methods, including thresholding, edge detection, or region-growing, often struggle to capture the fine details of the vascular network [5,16]. They are particularly prone to failure when faced with noisy or low-contrast images. These techniques are highly sensitive to image quality, requiring extensive manual tuning, which can still result in suboptimal outcomes in certain cases. For example, region-growing methods can fail to differentiate vessels from surrounding structures when intensity values overlap, while edge detection may struggle with discontinuities caused by noise or low contrast. Even advanced preprocessing techniques like the Frangi filter, which enhances vessel visibility by analyzing Hessian matrices at multiple scales, are limited by their reliance on predefined parameters and their inability to adapt to highly variable and noisy image data [17,18].

Modern deep learning approaches, such as U-Net, have led to significant improvements in segmentation accuracy [1,19,20]. However, these methods are not without limitations, particularly when applied to coronary computed tomography angiography (CTA) images. U-Net, despite its strong performance in general medical image segmentation tasks, struggles with the complex topology and small-scale details of coronary vessels. Its reliance on global features often leads to the loss of fine details, which are crucial for accurate vessel delineation. Furthermore, U-Net’s segmentation performance is significantly impacted by noise and low-contrast regions, which are common in CTA images. Recent advancements, such as transformer-based models like SegFormer and Swin UNet [21,22], have shown promise in addressing some of these limitations. By incorporating self-attention mechanisms, these models are better able to capture both global and local features, which improves the segmentation of complex structures like coronary vessels. However, these models are not without their own challenges. Transformer-based models tend to be more computationally expensive and require large amounts of data for effective training. Additionally, their performance can degrade in the presence of highly imbalanced or noisy datasets, which are common in medical imaging tasks like CTA segmentation. Another major limitation of deep learning-based approaches lies in their dependence on large annotated datasets for training [3,23]. Creating high-quality annotations for vascular imaging is a labor-intensive process requiring expert knowledge, and intra-observer variability can lead to inconsistent and unreliable training data. In small or imbalanced datasets, deep learning models are prone to overfitting, reducing their generalizability and robustness. This issue is particularly acute in CTA images, where the anatomical complexity of the vessels demands extensive and precise annotation.

Additionally, deep learning models can suffer from overfitting when trained on small datasets, reducing their generalizability. The high computational demands and extended training times further hinder the adoption of these techniques in resource-constrained environments [5,24]. Responding to the above concerns, traditional methods such as filtering-based pre-processing can be integrated with deep learning architectures and region-growing techniques to increase segmentation accuracy. For instance, the Frangi filter enhances vessel visibility by analyzing image Hessian matrices at multiple scales, making it the potential approach for vessel segmentation. Combining the Frangi filter with deep learning models can improve the feature extraction process, allowing networks to focus on more relevant visual information and to perform efficiently using large amounts of unlabelled data [17,18].

Integrating regional growth into deep learning allows the model to learn more efficiently from different datasets, improving its generalization and reducing the need for manual annotation [25]. While most applications focus on retinal vascular segmentation, vascular segmentation in coronary angiography images is a potential application for such a combination of techniques [26]. Therefore, automatic or semi-automatic segmentation of medical images is one of the most important topics in the field recently, as it can significantly reduce the dependency on manual annotation, simplify workflows, and increase the efficiency and accuracy of diagnosis [27,28,29].

**Figure 2:**
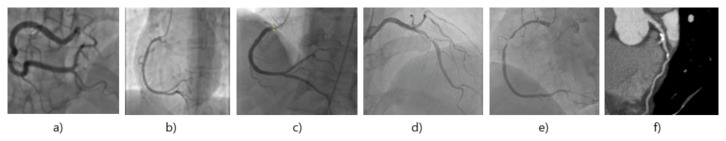
Visual comparison of ICA images (**a**) [26], (**b**) [30], (**c**) [15], (**d**) [2], (**e**) vs. CTA (**f**), emphasizing the high-resolution and detailed visualization typical of ICA, in contrast to the noise and contrast limitations of non-invasive CTA images.

However, accurate and automated segmentation is achieved when working with invasive coronary angiography (ICA) images, which are considered the gold standard for the diagnosis of obstructive coronary artery disease (CAD). These images are of high quality, allowing direct visualization of coronary artery anatomy, as presented in Figure 2a–d. Therefore, ICA images facilitate the efficient application of advanced computational techniques for high-precision segmentation and stenosis assessment [30]. Despite its invasive nature and associated risks, such as complications from catheter insertion and radiation exposure, ICA, X-ray angiography (XRA), or X-ray coronary angiography (XCA) remains popular in both clinical practice and research, particularly in patients with significant symptoms or high risk of adverse cardiovascular events [2,15,26]. On the other hand, CTA is a non-invasive imaging modality popular for its safety and speed, which is beneficial for initial evaluations in patients with a complex risk profile, as it allows rapid diagnosis and management decisions while minimizing patient discomfort. In addition, CTA is effective in reducing the incidence of major adverse cardiovascular events when used as a first-line diagnostic tool. However, segmenting blood vessels in CTA is considerably more challenging than in ICA due to the complex and variable geometry of coronary arteries, along with image quality issues and noise, as displayed in Figure 2f. The complex topology of the coronary arteries, with tortuous pathways and multiple branches, makes accurate tracking and prediction difficult. Such images are often affected by noise and partial volume effects, which make it difficult to distinguish blood vessels from surrounding tissue.

Despite these challenges, the scientific interest in developing effective segmentation techniques for CTA remains high. And since CTA is increasingly preferred in many clinical scenarios, it is reasonable to explore the possibilities of advanced AI methods, particularly those utilizing deep learning approaches, for segmenting blood vessels and their calcified plaques in CTA images.

Although CTA images are safer and more accessible, their accurate segmentation is challenging due to noise, varying contrast, and complex coronary geometry. However, CTA is a relatively common choice in clinical practice, and the development of accurate segmentation techniques is needed to facilitate clinician workflow and to improve the accuracy of disease diagnosis. This study aims to address these gaps by using deep learning and preprocessing techniques to improve the segmentation accuracy of CTA images, with a focus on overcoming noise, handling complex vascular geometry, and reducing the dependency on manual annotation.

The rest of this paper is organized as follows: Section 2 provides an overview of related work on vascular segmentation methods, distinguishing between traditional methods and deep learning approaches. Section 3 presents the proposed solution, including the DUCK-Net architecture and preprocessing techniques such as Frangi filtering and region growing. Section 4 describes the composition of the dataset, annotation tasks, and evaluation metrics. Section 5 discusses the experimental results, including segmentation accuracy and the effect of intra-observer variability and segmentation issues on calcified plaque. Finally, Section 6 concludes the study, summarizes the findings, includes a discussion of the calcified plaque segmentation task, and identifies directions for future research.

## 2. Deep Learning for Coronary Artery Segmentation in CTA Images

Recent advances in deep learning architectures and attention mechanisms have greatly improved the performance of segmentation tasks, particularly in medical images [31]. In the context of cardiac angiography, deep learning can enhance the detection of coronary artery disease by accurately interpreting intricate patterns within the images [32,33]. Despite its potential, deep learning in medical imaging faces challenges, in particular in ensuring access to high-quality annotated datasets, which are essential for training effective models, but are often limited in clinical settings. A comparison of different deep learning architectures for coronary segmentation is provided in Table 1.

Further research should therefore focus on developing unsupervised, or semi-supervised or few-shot learning techniques to reduce dependence on labeled data, and integrating multi-modal data (e.g., fusion of magnetic resonance imaging (MRI) and computed tomography (CT) images) in order to improve segmentation accuracy. Alternatively, as an intermediate option, models can be developed by integrating weakly supervised learning, which is used to address the problem of data scarcity, where a small amount of labeled data are used in combination with a larger amount of unlabeled data, so that models can be trained efficiently and generalize better even with a limited number of labeled examples.

Cardiac image segmentation datasets, including the Automated Segmentation of Coronary Arteries (ASOCA) challenge [43], ACDC (Automated Cardiac Diagnosis Challenge) [44], MM-WHS (Multi-Modality Whole Heart Segmentation) [45], and others, are typically provided through organized challenges aimed at benchmarking algorithms under standardized conditions. However, these datasets tend to be small, reflecting the complexity and cost of generating high quality annotated medical data. This limitation is possibly one of the reasons why the average Dice score coefficient (DSC) remains around 0.85, as documented in Table 1, suggesting that the segmentation results can still be improved.

## 3. Materials and Methods

In response to the challenges of blood vessel segmentation, this study focuses on the analysis of CTA images, where we need to detect and segment blood vessels in order to subsequently detect and segment calcified plaques. The presence of calcified plaque is a key indicator of coronary artery disease, providing critical information about the degree of arterial blockage and the associated risk of cardiovascular events. Proper segmentation of blood vessels and plaques can significantly aid in the diagnostic process.

For the segmentation task, we employed the state-of-the-art DUCK-Net architecture [46], which has demonstrated superior performance in medical image segmentation, as validated on the Kvasir-SEG benchmark. DUCK-Net builds on the widely-used U-Net architecture, incorporating significant enhancements to improve its segmentation capabilities.

**Figure 3:**
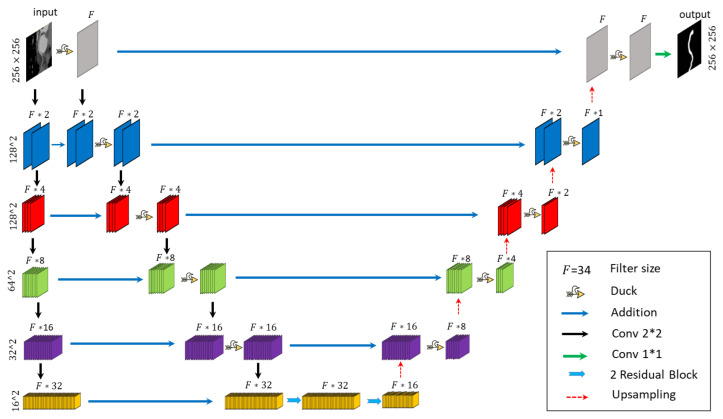
DuckNet Architecture Overview.

A key innovation in DUCK-Net is the introduction of the DUCK block at each stage of the network, except for the final layer. The DUCK block employs six different variations of convolutional blocks in parallel, allowing the network to automatically select and train the most effective block for a given task. This adaptive capability enhances the network’s flexibility, enabling it to handle the complex variations in vessel structures and image noise found in medical data. By leveraging this parallel convolutional approach, DUCK-Net is able to learn more robust feature representations, leading to improved segmentation accuracy. Moreover, such improvements allow DUCK-Net to achieve better results when dealing with limited amounts of medical image data. DUCK-Net architecture is visualized in Figure 3.

To address the limited size of the dataset and to improve the generalization of the model, we have also applied a number of advanced data augmentation techniques tailored to image segmentation tasks, including geometric transformations, intensity adjustments and noise-based augmentations. These augmentations aimed to introduce variability in the training data, allowing the model to better adapt to different vessel structures. Furthermore, denoising plays a crucial role in medical image processing as it enhances image quality by reducing noise, resulting in clearer visualization of anatomical structures and better performance of subsequent analysis and segmentation tasks [47,48,49].

### 3.1. Dataset

#### 3.1.1. Dataset Composition

The dataset used in this research consists of images from 48 patients, with each patient having three primary blood vessels—Left Coronary Artery (LCA), Right Circumflex Artery (RCX), and Left Anterior Descending Artery (LAD)—represented in nine images per vessel. In total, the dataset consists of 1296 annotated images.

The images were manually annotated by a small group of radiologists using a mouse as the input device, with the option to zoom in on the images during the annotation process [50]. Due to data sensitivity, it was decided to self-host the solution. This tool offers the advantage of requiring no software installation or user registration. Because of the tool’s limitations, annotations were initially exported in COCO format and subsequently converted into image-mask pairs for segmentation tasks. The dataset was then split into 75% training, 15% validation, and 10% test sets.

**Figure 4:**
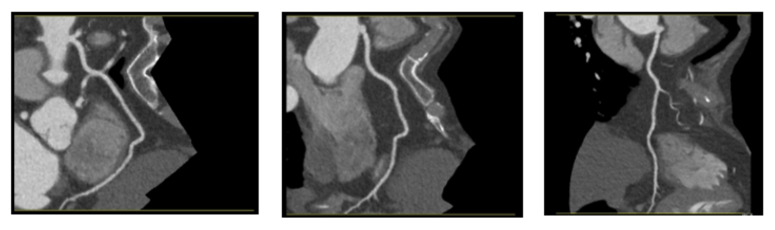
RCA vessel images from the same patient, highlighting variations despite identical vessel anatomy and patient characteristics.

The images have a consistent resolution of 512 × 512 pixels. After consultations with radiologists, it was decided to merge all images of the LCA, RCX, and LAD vessels, treating them as separate instances in the segmentation process to simplify the machine learning models task for blood vessel segmentation. Example images are provided in Figure 4.

#### 3.1.2. Annotation Issues

Approximately 16% of the images had DICE scores of up to 0.86 from the test data and presented the following challenges: selection of different blood vessel branches, variation in selected starting points, and inconsistency in annotated ending points. The greatest discrepancy occurs when blood vessels branch, as the model struggles to learn which path to follow. No instances of vessel branching were observed at the annotated locations.

**Figure 5:**
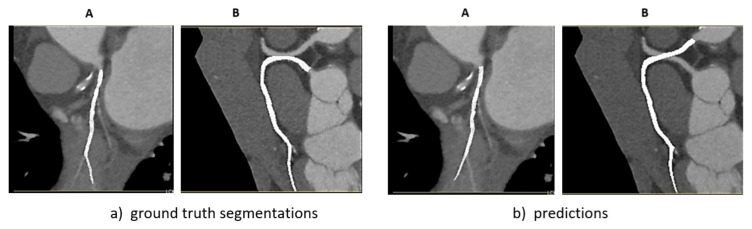
Examples of (**a**) ground truth and (**b**) model predictions for coronary artery blood vessel segmentations.

To assess intra-observer variability, radiologists were asked to re-annotate a subset of images from the test set. The purpose was to assess the consistency of the annotations over time and to identify possible discrepancies due to individual interpretation. The 11 selected images were re-annotated and analyzed to provide information on the robustness of the segmentation process and the impact of annotation variability on model performance. Cases where different branches were predicted are illustrated in Figure 5.

**Figure 6:**
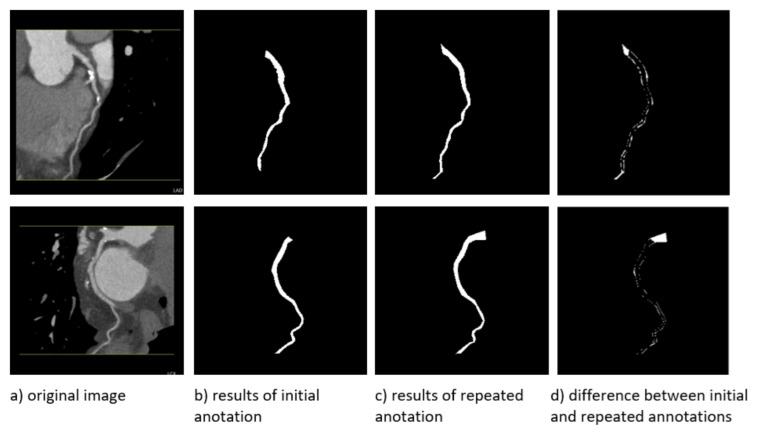
Instances of annotated vessels including initial and repeated annotation and their differences.

Another intra-observer common annotation issue is the inaccurate identification of the starting point of the blood vessel during training. This often leads to noticeable differences between the annotated and predicted segments, with minor discrepancies primarily observed along the edges, especially at the beginning of the vessel. As a result, these cases typically yield DICE scores ranging from 0.8 to 0.83. This can be observed in Figure 6b,c initial and repeated annotations. Despite the fact that this study has developed standardized annotation guidelines by including clear instructions on how to annotate blood vessels, the human error factor is still quite significant. Therefore, the best strategy to ensure consistency is based on stepwise annotation, where several levels of annotation are included to review and validate a subset of annotations. An annotation process with feedback mechanisms, as was the case in our study, is effective but requires additional work by both the IT team and the medical staff. However, automatic or semi-automatic annotation is the preferred solution. As an example, methods such as region growing can provide initial annotations that can be refined by annotators, thus reducing manual workload and variability.

In cases where the Dice score is above 0.85, minor inaccuracies along the edges still persist, and occasionally the segmentation does not extend to the bottom of the image. After discussions with specialists, it was determined that in these instances where the segmentation does not reach the bottom, the omission is deemed insignificant and does not adversely affect the overall analysis. This can be observed in Figure 6.

In Figure 6, the binary mask differences between the images are displayed. From the comparison of Figure 6d it is evident that the discrepancies are primarily located at the beginning and end of the blood vessel, as well as along the vessel walls. These types of inconsistencies are present throughout the dataset, mostly due to human error during the annotation process.

**Figure 7:**
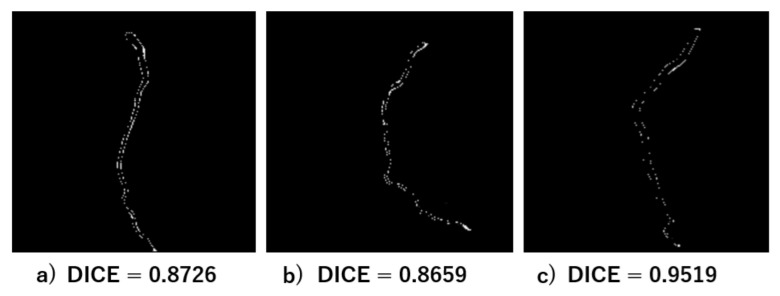
Boundary inaccuracies representing minor differences between the ground truth and predicted masks.

Figure 7 illustrates the white pixels representing differences between the ground truth mask and the predicted mask, highlighting minor discrepancies in segmentation results. Figure 7a,b shows the image examples with a Dice score of 0.8726 and of 0.8659, respectively. The best result from the test data achieved a Dice score of 0.9519 (Figure 7c).

The discrepancies are mainly due to inaccuracies around the edges of the dishes, resulting in scores ranging from 0.87 to 0.95.

Regarding the typical errors mentioned earlier, the annotation of the upper heart vessels is not always consistent, leading to ambiguity about how far the segmentation should extend. However, this may not be a critical issue. In some instances where vessel branching occurred, a different path was selected during annotation. This discrepancy is illustrated in Figure 5.

### 3.2. Proposed Solution

Our proposed solution leverages the previously mentioned 2D dataset and employs the state-of-the-art DUCK-Net network architecture for effective segmentation of blood vessels in CT images. The DUCK-Net architecture, recognized for its innovative design, enhances feature extraction and improves segmentation accuracy, making it particularly suitable for medical imaging tasks.

In addition to utilizing DUCK-Net, we incorporate a region-growing technique to effectively filter out noise from the CT images. This method identifies and groups neighboring pixels with similar intensity values, allowing us to focus on relevant anatomical structures. By applying region growing, we can significantly simplify the input images, which not only reduces the complexity of the data but also facilitates a faster convergence of the model during training. With cleaner input data, the DUCK-Net architecture can learn more efficiently, ultimately leading to improved segmentation performance.

**Figure 8:**
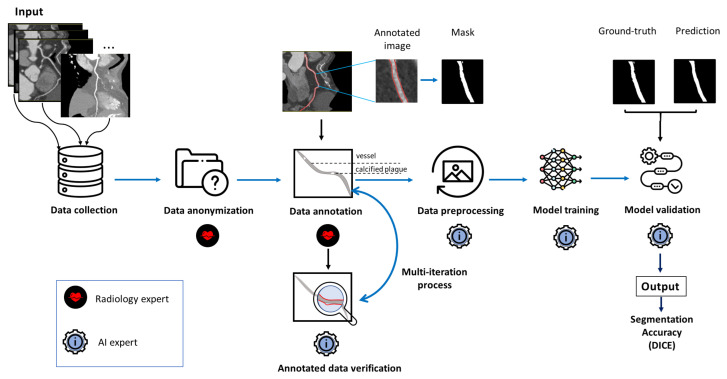
The pipeline of the proposed segmentation solution.

Given the challenges posed by intra-observer variability in annotating such datasets, we propose a solution (Figure 8) aimed at streamlining the annotation process. Instead of requiring extensive annotations for each image, our approach suggests that specialists annotate only the beginning of each blood vessel, and the end if applicable. This method not only simplifies the annotation task but also significantly reduces the time required to complete it. By minimizing the amount of detailed annotation needed, we can decrease the likelihood of errors that commonly arise during the annotation process, ultimately leading to more consistent and reliable training data.

The pipeline begins with data collection, where radiologists extract images from an internal database. These images are de-anonymized using a script that removes metadata fields from the DICOM format files, thus ensuring privacy, before they are passed on to the AI development team. After a brief workshop on the annotation process, specialists label the images, and their annotations are then exported in COCO JSON format. Radiologists annotate the anonymized images, marking vessels and calcified plaques. These annotations are used to generate masks. A feedback loop between radiology and AI experts allows for the verification and refinement of the annotations, ensuring their quality Subsequently, a variety of pre-processing techniques are applied, including thresholding, Frangi filtering, region filling, etc. Below is the Pseudo-code Algorithm 1 for data pre-processing integrating thresholding, Frangi filtering, and region filling for vessel segmentation in 2D images. The algorithm starts with thresholding (the first step), where the 2D input image is normalized and the global threshold *T* with a value of 0.47 is applied to create a binary mask. Next, Frangi filtering is applied to enhance tubular structures by analyzing the Hessian matrix across multiple scales and computing a vesselness measure based on eigenvalues, which highlights the vessels while suppressing non-tubular structures. A region-filling step is then used to identify the connected components in the mask, leaving only those components that meet the minimum size criterion and discarding smaller ones as noise. Finally, the results of thresholding and region-filling, combined with Frangi filtering, are merged into a single mask, which can be further improved by morphological operations to fill gaps and smooth edges. The final result is the segmented vascular map.

The model is trained repeatedly using each pre-processing technique to identify the most effective approach. Validation is conducted using a separate dataset that remains unseen by the model during training. The final segmentation results are compared with the ground truth masks to evaluate the model’s accuracy. The output is represented in terms of segmentation accuracy, primarily using the Dice coefficient.
**Algorithm 1** Pre-processing of vessel images**Input**: 2D Image I(x,y), scales $\left[\sigma_{min},\sigma_{max}\right]$, step size Δσ, threshold *T*, parameters α,β**Output**: final segmented vesselness map S(x,y)**1: Thresholding**   a. Normalize the image intensity to [0,1];   b. Apply global threshold *T*: $T_{mask}\left(x,y\right)=1$, if $I_{norm}\left(x,y\right)\geq T$, else 0.**2: Frangi Filtering**   a. Initialize vesselness map V(x,y)=0 for all pixels;   b. For each scale σ in range $\left[\sigma_{min},\sigma_{max}\right]$, with step Δσ:         b.1. Smooth the image using a Gaussian filter: $I_{\sigma }=I\left(x,y\right)*G\left(x,y,\sigma \right)$;         b.2. Compute second-order derivatives $I_{xx}$, $I_{xy}$, $I_{yy}$;         b.3. Construct the Hessian matrix:          $$H=\left[\begin{array}{cc} I_{xx} & I_{xy}\\ I_{xy} & I_{yy} \end{array}\right]$$         b.4. Compute eigenvalues $\lambda_{1},\lambda_{2}$ of *H* ($|\lambda_{1}\left|\leq \right|\lambda_{2}|$);         b.5. Compute vesselness measures:            $BR=\frac{|\lambda_{1}|}{|\lambda_{2}|}$, (blobness ratio); $S=\sqrt{\lambda_{1}^{2}+\lambda_{2}^{2}}$ (structuredness)         b.6. Compute vesselness response:
$$V_{\sigma }\left(x,y\right)=\left\{\begin{array}{ll} 0, & \mathrm{if}\;\lambda_{2}>0\\ \exp\left(-\frac{RB^{2}}{2\alpha^{2}}\right)\cdot \left(1-\exp\left(-\frac{S^{2}}{2\beta^{2}}\right)\right), & \mathrm{otherwise} \end{array}\right.$$         b.7. Update vesselness map: $V\left(x,y\right)=\max(V\left(x,y\right),V_{\sigma }\left(x,y\right))$;         b.8. Threshold vesselness map:$$V_{\mathrm{mask}}\left(x,y\right)=\left\{\begin{array}{ll} 1, & \mathrm{if}\;V\left(x,y\right)\geq T_{\mathrm{vesselness}}\\ 0, & \mathrm{otherwise} \end{array}\right.$$**3. Region Filling**:   a. Identify connected components in $V_{mask}$;   b. **for** each connected component:            **if** size of the component ≥min_size, **then** retain the component;            **else** discard the component as noise;            **end if**   c. Fill gaps using morphological closing: $F_{mask}$ = MorphClosing($V_{mask})$**4. Combine Results:**      a. Combine thresholded image and Frangi-filtered results:$$C_{mask}\left(x,y\right)=T_{mask}\left(x,y\right)\vee \{F_{mask}\left(x,y\right)$$**5. Final Output:**      a. Apply post-processing      b. Return final segmented vessel map: $S\left(x,y\right)=C_{mask}\left(x,y\right)$

## 4. Experimental Results

### 4.1. Metrics

Multiple experiments were conducted to evaluate the performance of our proposed segmentation techniques, and the results were subsequently compared using the Dice score coefficient, which measures the overlap between the predicted segmentation and the ground truth.

(1) $$\mathrm{DSC}=\frac{2|X\cap Y|}{\left|X|+|Y\right|}=\frac{2\cdot \mathrm{TP}}{2\cdot \mathrm{TP}+\mathrm{FP}+\mathrm{FN}}$$

- *X*: The set of pixels in the predicted segmentation.
- *Y*: The set of pixels in the ground truth segmentation.
- |X|: The number of elements (pixels) in the predicted segmentation.
- |Y|: The number of elements (pixels) in the ground truth segmentation.
- |X∩Y|: The number of elements common to both sets (the intersection of the predicted and ground truth segmentations).

In the first attempt (A), the analysis was performed using the originally provided CT images in conjunction with their corresponding annotated masks. This baseline allowed us to establish a reference point for evaluating the effectiveness of our region filling techniques.

The second attempt (B) involved the application of a basic region filling technique. This approach aimed to improve segmentation results by effectively addressing noise and enhancing the continuity of the vessel structures. Notably, this method does not require any additional annotations during inference.

The third attempt (C) incorporated a preprocessing step using the Frangi filter, which is well-known for its ability to enhance tubular structures. By applying this filter to the input images, we aimed to accentuate the vascular features before proceeding with segmentation.

**Figure 9:**
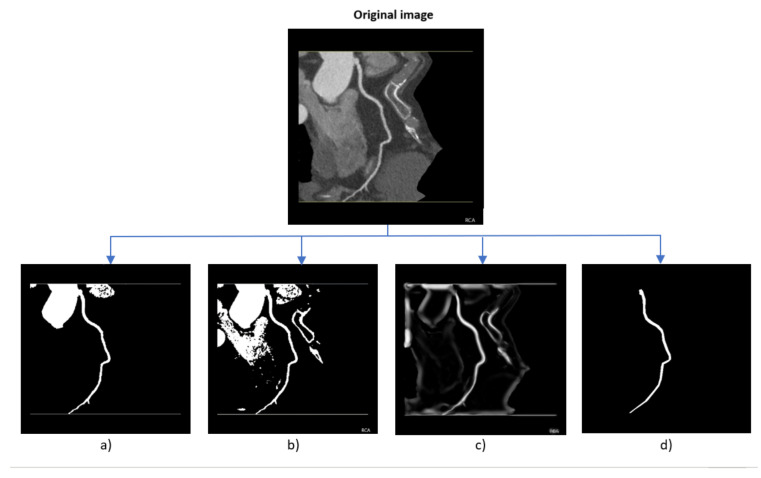
Original image with (**a**) applied threshold, (**b**) region fill and threshold, (**c**) applied Frangi filter and (**d**) ground truth segmentation.

Visualized examples can be seen in Figure 9.

Finally, the fourth attempt (D) utilized an advanced region filling technique, which incorporated more sophisticated algorithms to capture the intricate details of the vascular structures more accurately. This method aimed to leverage contextual information from the surrounding pixels, providing a more robust segmentation outcome. However, it requires the radiologist to select a reference point within the blood vessel to initiate the inference process.

Results of these attempts are presented in Table 2.

The radiologists were tasked with identifying both the best and the worst images. However, the variations were so minor that the differences between their annotations and predictions were deemed clinically insignificant and considered equivalent to the best human annotations. As a result, these images could be treated as equivalent in clinical practice for further medical analyses, such as evaluating the severity of stenosis.

After reviewing various state-of-the-art models, we observed that most of them incorporate the U-Net architecture with different modifications. Swin-Unet, a transformer-based model, demonstrated significantly better performance compared to the standard U-Net. One notable observation is that Swin-Unet reached the convergence point at around 370 epochs, which is faster than U-Net (490 epochs), Duck-Net (430 epochs), and our proposed solution (410 epochs). As indicated in Table 3, the progression of the Dice metric clearly illustrates performance improvements: starting with the U-Net as the worst performer, followed by improvements in Swin-Unet, further enhancements in Duck-Net (achieving a Dice of 0.8978), and finally reaching 0.9108 Dice with our proposed solution.

Statistically significant performance differences between models, U-Net, Duck-Net, Swin-U-Net, and Morpho-U-Net, are provided in Table 4 where significant differences (p<0.05) are bolded. The Morpho-U-Net showed notable performance differences compared to the mentioned architectures, demonstrating its greater segmentation accuracy.

### 4.2. Comparison of Intra-Observer Annotations

To address the previously noted annotation issues, radiologists were tasked with re-annotating the same images they had previously annotated.

After updating the annotations selected from the test set, new DICE values were calculated as follows:

- Prediction against previously annotated data (old)
- Prediction against newly annotated data (new)
- Difference between previously annotated and newly annotated data (human)

As observed in Table 5, the newly acquired annotations were annotated with greater precision. This was evident both visually, by examining the differences between predictions and previously annotated images, and quantitatively through the DICE metric. These repeated annotations demonstrated inter-observer variability when annotating this dataset. The minimum overlap between annotation rounds was 0.6901, while the highest similarity between annotations yielded an overlap of 0.8985.

The considerably higher accuracy observed in the “New” annotations suggests that, given the variability in annotation, it is more effective to focus on accurately annotating a smaller set of images rather than a large volume. With a sufficient number of high-quality annotations, the model becomes more accurate and consistent, enabling it to annotate blood vessels without supervision.

**Figure 10:**
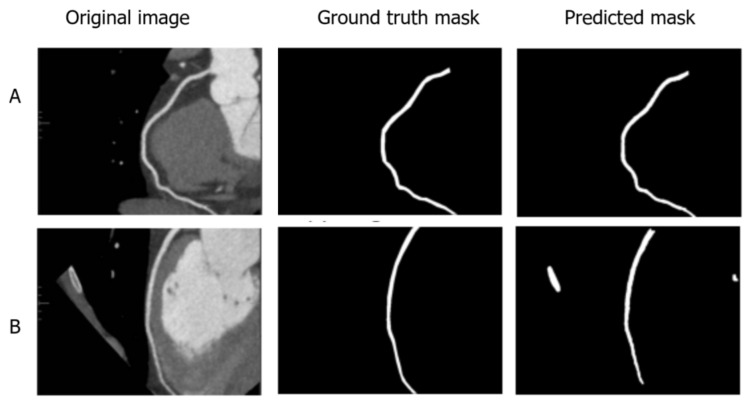
Examples of segmentation results using Morpho-U-Net, resulting in DICE values of 0.927 for image (**A**) and 0.759 for image (**B**).

Additional experiments have been made using the standard U-Net network, and extended U-Net model, named Morpho-U-Net, which enhances traditional architecture by integrating advanced morphological operations to improve vessel segmentation. Results can be observed in Figure 10. U-net requires significantly more epochs to accurately recognize blood vessels, often interpreting them as a continuous mask. The model can generally identify blood vessels with correct shapes, the segmented vessels tend to appear narrower than the ground truth, resulting in an average DICE value of 0.722. In Morpho-U-Net, the image is first smoothed using Gaussian blur, followed by thresholding to generate a binary mask. Next, morphological opening is applied, involving erosion followed by dilation to remove small noise. This is followed by morphological closing, which applies dilation first and then erosion to fill gaps and refine the vessel structures, this can be seen in Figure 10. This approach allows removing unwanted noise, closing the gaps in the blood vessel in order to make it continuous improving the DICE value by 9% resulting in an average of 0.829.

## 5. Discussion

Due to hardware and time limitations, we trained and tested the model on 256 × 256 resolution instead of the provided 512 × 512 images in the dataset. By increasing the resolution, we expect the coronary artery to consist of twice as many pixels in its width, which could further improve accuracy metrics. This is because having predictions off by even a single pixel on one or both sides can significantly impact accuracy. The next step in our work could involve modifying the solution to handle larger or dynamic resolutions and revalidating it. Following the blood vessel segmentation, we plan to detect and localize plaques, using the segmented images to calculate the extent of blood flow restriction.

Dataset analysis revealed the following counts: 1246 blood vessels, with 704 instances of non-calcified plaque, 1144 instances of mixed plaque, and 320 instances of calcified plaque. While these counts may initially appear imbalanced, the number of calcified plaques is sufficient for training an accurate model due to their brightness and blooming artifacts, which make them easier to detect. Mixed and non-calcified plaques are relatively balanced. The model achieved a recall of approximately 0.75, indicating reliable localization of plaques but showing less precision in boundary detection.

An approach to crop out regions outside of blood vessels was attempted but abandoned, as calcified plaques proved easier to detect and segment, while nearly 50% of non-calcified plaques were annotated outside the blood vessel boundaries. This led to poor results. Another challenge was that the model detected more calcified plaques than annotated, raising concerns about the ground truth quality. This discrepancy could be due to human error, incomplete annotations, or possibly superior model performance in localizing calcified plaques.

Detecting mixed and non-calcified plaques posed additional difficulties, as these structures tend to have darker pixels compared to the bright white of calcified plaques. Blood vessels often contain surrounding dark pixels, suggesting a need for an alternative approach with a different training network, a larger and higher-quality dataset, and possibly a revised segmentation strategy.

**Figure 11:**
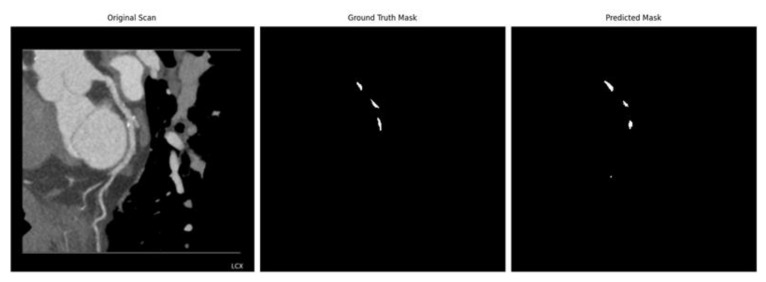
Segmentation results for calcified, mixed, and non-calcified plaques.

The dataset was also annotated for plaques, with classes including calcified, non-calcified, and mixed plaques. Examples of such classes can be seen in Figure 11. Calcified plaques proved to be the easiest to segment due to their higher contrast in imaging. Segmenting non-calcified and mixed plaques presented greater challenges. These difficulties likely stemmed from their lower contrast, making them harder to distinguish from the surrounding background.

Initially, it was decided to crop the images based on the blood vessel contours. However, a preliminary analysis revealed that, on average, 85.07% of the total area of calcified plaques was contained within the blood vessel boundaries, whereas only 49.83% of the area of non-calcified plaques fell within these limits. Omitting the data outside the blood vessel boundaries would result in the model losing critical contextual information necessary for accurately identifying non-calcified plaques.

**Figure 12:**
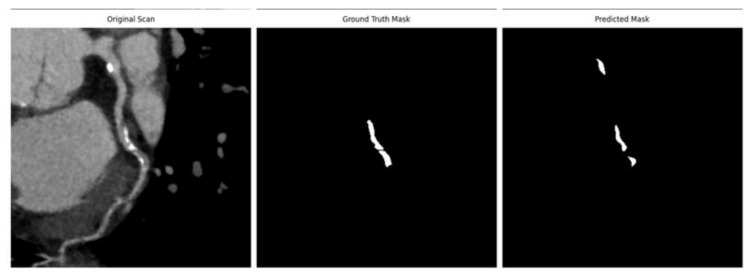
Examples of incomplete annotations.

The accuracy, measured using the Dice metric, is equal to 0.88 for calcified plaques, 0.65 for mixed plaques, and 0.43 for non-calcified plaques. This discrepancy in performance can be partly attributed to the quality of the dataset. Some images were not fully annotated for all three classes, leading to potential confusion during the training process, this can be observed in Figure 12. Despite these challenges, the visual results are promising, as the model has demonstrated the ability to detect additional plaque instances that were missed in the annotation process.

## 6. Conclusions

This study highlights the complexities and advancements in blood vessel segmentation using deep learning techniques. It demonstrates that the proposed region filling and Frangi filter techniques significantly enhance the accuracy of vessel segmentation in angiographic images. The classical U-Net model, with an average Dice score of 0.722, requires many training epochs, and the segmentation results tend to indicate narrower blood vessels compared to the ground truth. The use of Morpho-U-Net incorporating advanced morphological operations such as Gaussian blurring, thresholding and morphological opening/closing improves the integrity of the blood vessels and reduces noise, resulting in a higher Dice score of 0.829. In addition, the study highlights that intra-observer variability—differences between annotations made by the same expert at different times—can lead to inconsistencies, which affect the performance of the model. To overcome this problem, it is apparent that the focus should be on building smaller, high quality datasets with consistent annotations, rather than on building large datasets with variable quality.

Future research should concentrate on improving DUCK-Net’s resilience and generalizability by using semi-supervised learning strategies. The lack of annotated medical data can be addressed by semi-supervised frameworks that can use unlabeled data in addition to a small amount of labeled data. This method can greatly reduce the workload associated with human annotation, while facilitating the creation of more precise and effective models. Furthermore, by applying knowledge from related medical imaging tasks, poorly supervised learning or transfer learning may be investigated to further improve performance in small datasets.

Furthermore, the model needs to be improved to segment plaques and evaluate their attributes. The clinical value would be enhanced by expanding to the multi-class segmentation of calcified, mixed, and non-calcified plaques. It makes sense to incorporate plaque segmentation into a pipeline for automated risk assessment and stenosis evaluation. Last but not least, extending the capabilities of the model to handle high-resolution images or to dynamically adapt to different resolutions will ensure its wider usefulness in a clinical context.

## Figures and Tables

**Table 1:** Comparison of different deep learning models for coronary artery segmentation.

Source	DSC	Data	Model
[34]	0.86	CorArtTS2020 dataset of 81 cases	DR-LCT-UNet
[35]	0.73	25 manually corrected segmentations	I2I-3D
[36]	0.89	69 subjects with chest pain ( data pre-processed using 3D Slicer; 14597 slices)	U-net++ [Xnet/BN]
[37]	0.90	CTA dataset of 20 patients	FCN, FCN-AG1, FCN-AG2 fusion.
[38]	0.86	CTA dataset of 30 patients	UNet
[39]	0.65	98 multi-vendor ECG-gated cardiac CT scans	nnU-Net
[40]	0.83	60 cases of CTA images (ASOCA challenge)	UTNet
[41]	0.78	60 cases of CTA images (ASOCA challenge)	CoTr
[42]	0.86	60 cases of CTA images (ASOCA challenge)	Video Swin Transformer

**Table 2:** Dice Coefficients for Various Segmentation Approaches.

Attempt	Minimum	Maximum	25th Percentile	50th Percentile	75th Percentile
A	0.8746	0.9519	0.8999	0.9029	0.9074
B	0.8866	0.9531	0.9048	0.9108	0.9155
C	0.8643	0.9406	0.9001	0.9046	0.9086
D	0.8738	0.9510	0.8958	0.9071	0.9130

**Table 3:** Performance Metrics for Various Segmentation Approaches.

Approach	Dice	Precision	Recall
Morpho-U-Net (Ours)	0.9108	0.9341	0.8872
Duck-Net	0.8978	0.9153	0.8691
Swin-Unet	0.8756	0.8871	0.8714
U-Net	0.7220	0.8925	0.8563

**Table 4:** Pairwise comparison matrix of segmentation models using *p*-values.

	Morpho-U-Net	Duck-Net	Swin-Unet	U-Net
Morpho-U-Net	-	0.044	0.036	0.031
Duck-Net	0.044	-	0.134	0.042
Swin-Unet	0.036	0.134	-	0.047
U-Net	0.031	0.042	0.047	-

**Table 5:** Comparison of Old, New, and Human DICE values across different statistical metrics.

	Average	Min	Max	25th Percentile	50th Percentile	75th Percentile
Old	0.8193	0.7525	0.8611	0.8006	0.8302	0.8403
New	0.8819	0.7531	0.9202	0.8849	0.9023	0.9066
Human	0.8146	0.6901	0.8985	0.7858	0.8061	0.8487

## Data Availability

The data and the code used for this study are available from the corresponding author upon reasonable request.
